# Exploring the molecular basis of adaptive evolution in hydrothermal vent crab *Austinograea alayseae* by transcriptome analysis

**DOI:** 10.1371/journal.pone.0178417

**Published:** 2017-05-26

**Authors:** Min Hui, Chengwen Song, Yuan Liu, Chaolun Li, Zhaoxia Cui

**Affiliations:** 1 Key Laboratory of Experimental Marine Biology, Institute of Oceanology, Chinese Academy of Sciences, Qingdao, China; 2 Laboratory for Marine Biology and Biotechnology, Qingdao National Laboratory for Marine Science and Technology, Qingdao, China; 3 National & Local Joint Engineering Laboratory of Ecological Mariculture, Qingdao, China; Xiamen University, CHINA

## Abstract

Elucidating the genetic mechanisms of adaptation to the hydrothermal vent in organisms at genomic level is significant for understanding the adaptive evolution process in the extreme environment. We performed RNA-seq on four different tissues of a vent crab species, *Austinograea alayseae*, producing 725,461 unigenes and 134,489 annotated genes. Genes related to sensory, circadian rhythm, hormone, hypoxia stress, metal detoxification and immunity were identified. It was noted that in the degenerated eyestalk, transcription of phototransduction related genes which are important for retinal function was greatly reduced; three crucial neuropeptide hormones, one molt-inhibiting and two crustacean hyperglycemic hormone precursors were characterized with conserved domains; hypoxia-inducible factor 1 and two novel isoforms of metallothioneins in the vent crabs were discovered. An analysis of 6,932 orthologs among three crabs *A*. *alayseae*, *Portunus trituberculutus* and *Eriocheir sinensis* revealed 19 positive selected genes (PSGs). Most of the PSGs were involved in immune responses, such as *crustins* and *anti-lipopolysaccharide factor*, suggesting their function in the adaptation to environment. The characterization of the first vent crab transcriptome provides abundant resources for genetic and evolutionary studies of this species, and paves the way for further investigation of vent adaptation process in crabs.

## Introduction

Deep-sea hydrothermal vents and their resident animals were first discovered in the 1970's after an extensive exploration along the Galápagos Rift [1–3]. Vents are well known for their challenging environment, such as low oxygen, lack of light, high hydrostatic pressure and thermal gradient, high levels of sulfide and heavy metals. One additional surprise of the vents is that they are unique ecosystems based on microbial chemoautotrophic production [4]. However, these seemingly toxic hydrothermal fluids directly support productive biological communities with a high proportion of endemic species [5,6]. Therefore, the organisms living in the hydrothermal vents have developed well adaptive mechanisms to survive in this extreme habitat.

Many invertebrates dominate the hydrothermal vents and decapod crustaceans occupy approximately 10% of all taxa identified from these sites [7,8]. Among them, six genera (*Allograea*, *Austinograea*, *Bythograea*, *Cyanagraea*, *Gandalfus* and *Segonzacia*) of brachyuran crabs have been found and all belong to a single family, Bythograeidae Williams, 1980. They are the most common and abundant species, and predators at the top of food chain in the vent ecosystem [9]. As living in the extreme environment, the vent crabs are characterized with specific morphological traits such as whole white body, and reduced eyestalks with vestigial cornea. Therefore, exploring the gene profile and determination of the selective force that drive molecular evolution of the vent crabs are fundamental to understand the genetic basis of the adaptation to the hydrothermal vent environment.

Most studies for the vent crabs are mainly focused on the complete mitochondrial genome sequences [10,11] and the molecular systematics of the vent species [9,12,13]. There is currently limited genomic resource for the vent crabs as well as other decapods, although it is significant to reveal the mechanism of adaptation to the vent. The cDNA sequences of *metallothionein* (*MT*) genes have been obtained and characterized in three species of the Bythograeidae, and in spite of the unique environmental conditions, no specific amino acid residue for the MT of the vent crabs has been found [14]. By the subtractive suppression hybridization experiments in *G*. *yunohana* and *P*. *trituberculatus*, 51 transcripts with annotations have been identified to be differentially expressed, and theoredoxin (Trx) and heat shock protein 90 (Hsp90) have been primarily analyzed [15]. However, the information at the transcriptome level of the vent crabs is far from sufficient.

The development of next generation sequencing (NGS) technology and bioinformatics provides a global perspective on taxonomic profiling of genes expressed under the influence of different environmental conditions where these organisms live [16], and the coordinated transcriptional changes in multiple genes that involve in various biological pathways [17]. For instance, recent high-throughput transcriptomic studies have been performed in the vent and cold seep mussels (*Bathymodiolus azoricus* and *B*. *platifrons*), respectively [18,19]. An extensive collection of transcripts have been obtained, and many genes related to innate immunity, heavy metal detoxification and sulfide metabolism have been identified. It can serve as basis for future molecular studies on extreme environment adaptation, including the host-symbiont interaction mechanism in mussels. To date, no such gene profile in a large scale has been explored in any crustaceans living in deep-sea hydrothermal vents.

The objective of this study is to generate a comprehensive transcriptome database (including eyestalk, gill, hepatopancreas and muscle) for the hydrothermal vent crab, *A*. *alayseae* Guinot, 1990 and clarify the intertwined gene regulatory mechanism for the vent adaptation preliminarily. Candidate genes that may facilitate inhabiting in the vent environment have been identified. By orthologous gene comparison with two shallow-water crab species (*P*. *trituberculutus* and *E*. *sinensis*), genes that might have experienced positive selection among the crabs have been explored. This is the possible inner driving force for the vent crab adaptive evolution.

## Materials and methods

### Ethics statement

The experiments were conducted in strict accordance with the guidelines of the Institutional Animal Care and Use Committee (IACUC) of the Chinese Academy of Sciences (No. 2011–2). This study was specifically approved by the Committee on the Ethics of Animal Experiments of the Institute of Oceanology at the Chinese Academy of Sciences. All efforts were made to minimize the suffering of the crabs.

### Sample collection

Two deep-sea crabs *A*. *alayseae* (one male and one female) were collected from the South China Sea (151°52′50.084″E, 3°42′47.259″S) with a depth of 1995 m and temperature of 1.01°C on June 10, 2015. Four different tissues (eyestalk, gill, hepatopancreas and muscle) from each individual were sampled in order to obtain as many expressed genes as possible. After dissection, the samples were immediately frozen in liquid nitrogen for RNA extraction.

### Illumina sequencing and *de novo* assembly

The total RNA from the eight samples was extracted using the TRIzol kit (Invitrogen, USA) according to the manufacturer’s protocol. For each sample, 1.5 μg RNA was prepared for RNA-Seq. Eight libraries for the tissues were constructed using NEBNext® Ultra™ RNA Library Prep Kit and sequenced on an Illumina HiSeq 2500 platform following the manufacturer’s instructions (Illumina, USA) and paired-end reads were generated. All raw sequences were deposited at NCBI Short Read Archive (SRA) database (http://www.ncbi.nlm.nih.gov/Traces/sra/). Clean reads were obtained by removing reads containing adaptor, reads containing ploy-N (the ratio of ‘N’ to be more than 10%) and low quality reads (the ratio of base pairs with quality score < 5 more than 50%). Transcriptome assembly was accomplished based on clean reads using Trinity [20] with min_kmer_cov set to 2 and all other default parameters. The longest copy of redundant transcripts was regarded as a unigene.

### Gene functional annotation and expression analysis

All the assembled unigenes were searched against the NCBI Nr (non-redundant protein sequences), Nt (non-redundant nucleotide) and Swiss-Prot (http://www.ebi.ac.uk/uniprot/) databases for gene annotation with an E-value cutoff of 1E-5. Genes encoding protein domains were identified by searching against Protein Family (Pfam) database (http://pfam.janelia.org/; E-value < 0.01). After that, GO (Gene Ontology; http://www.geneontology.org/; E-value < 1E-6) and KOG (euKaryotic Ortholog Group; http://www.ncbi.nlm.nih.gov/COG/; E-value < 1E-3) annotations were performed to classify the potential functions of the unigenes based on known orthologous gene products. GO enrichment analysis was implemented by the GOseq R packages, in which gene length bias was adjusted [21]. Biochemical pathway information of the unigenes was collected from KEGG (Kyoto Encyclopedia of Genes and Genomes; http://www.genome.jp/kegg/; E-value < 1E-10), and KOBAS software was used to test the statistical enrichment of unigenes in KEGG pathway [22]. To estimate gene expression levels, the clean reads of each sample were mapped to the assembled transcriptome to obtain read counts for each gene using RSEM [23]. The read counts were normalized to fragments per kilobase of exon model per million mapped reads (FPKM).

### Candidate gene discovery and sequence analysis

According to the annotation mainly from NR and Swiss-Prot databases, the genes with functions we concerned were manually checked by further searching UniProt database (http://www.uniprot.org/) as well as published literatures. The searched genes were related to sensory, circadian rhythm, neuropeptide hormone, immune system, chemical stress and metal transporter. SignalP 4.1 Server (http://www.cbs.dtu.dk/services/SignalP/) was used to predict the signal peptide. The multiple sequences alignment and the neighbor-joining (NJ) phylogenetic tree were conducted with MEGA 6.0 [24] based on amino acid sequences. Functional domain analysis was performed with SMART (http://smart.embl.de/).

### Orthologous genes identification and phylogenic tree construction

According to Williams, vent crabs Bythograeidae exhibit certain characters of the family Portunidae, and resemblance to the freshwater Potamidae [25]. Therefore, we also sequenced the transcriptomes of four tissues (eyestalk, gill, hepatopancreas and muscle) in two shallow water crabs, *E*. *sinensis* and *P*. *trituberculatus* (unpublished). The comparison among species was expected to reveal the genetic basis of the vent adaptation in *A*. *alayseae*. The CDS of each putative unigene was extracted according to the BLASTX results, and ESTScan software [26] was used to determine the direction of sequences that did not have align results. The resultant CDS extracted from each unigene was translated into amino acid sequences with the standard codon table. Reciprocal BLASTP was conducted for all amino acid sequences with a cut-off E-value of 1E-5. Orthologous groups were constructed with OrthoMCL v2.0.3 using default settings [27]. The one-to-one orthologous genes alignment was performed by MUSCLE based on protein sequences [28]. The phylogeny tree was constructed using PhyML [29] and visualized by MEGA6.0 [24].

### Positive selected genes identification

Ka/Ks ratio was estimated with PAML [30] package using default settings. Orthologous genes with a high Ka/Ks ratio were usually supposed to be evolving under positive selection, while genes with a Ka/Ks close to zero mainly had synonymous substitutions, indicating that these genes were under purified selection and conserved [17]. In this study, we defined genes with Ka/Ks > 1 as positively selected genes (divergent genes) and genes with Ka/Ks < 0.1 as conserved genes. In addition, GO and KEGG enrichment pathway analyses were also used to determine gene functional categories of divergent and conserved genes.

## Results and discussion

### *De novo* assembly, gene annotation and expression

A total of 591,265,622 raw reads for the vent crab transcriptomes were obtained. All raw data were deposited in the NCBI Sequence Read Archive repository (accession number: SRR4416314). After filtering, 75.13 Gbp clean data were remained. Then 864,469 transcripts and 725,461 unigenes were assembled with the N50 length of 423 bp for transcripts and 506 bp for unigenes. Detailed information of the sequence data was summarized in Table 1. Compared with other transcriptomic study for vent species, the average length of the transcripts here is shorter [18], which might be due to the slight RNA degradation of the sampled tissues. However, the large sequencing data should ensure the complete gene information obtained.

**Table 1:** Summary statistics of RNA sequencing data from *Austinograea alayseae*.

Assembly and Annotation	
Number of transcripts	864,469
Mean length of transcripts	414
N50 of transcripts	423
Number of unigenes	725,461
Mean length of unigenes	470
N50 of unigenes	506
Annotated in NR	32,056 (4.41%)
Annotated in NT	16,559 (2.28%)
Annotated in KO	36,810 (5.07%)
Annotated in Swiss-Prot	59,339 (8.17%)
Annotated in Pfam	107,290 (14.78%)
Annotated in GO	109,411 (15.08%)
Annotated in KOG	46,862 (6.45%)
Annotated in all databases	2,999 (0.41%)
Annotated in at least one database	134,489 (18.53%)

Based on different databases, 134,489 (18.53%) unigenes were finally annotated in at least one database (Table 1; S1 Table). All these genes were divided into 56 subcategories in GO analysis, mainly including ‘cellular process’, ‘metabolic process’, ‘single-organism process’, ‘cell’, ‘cell part’, ‘binding’ and ‘catalytic activity’ (S1 Fig). In KOG analysis, the main function classifications were found to be ‘General function prediction only’, ‘Signal transduction mechanisms’, ‘Posttranslational modification, protein turnover, chaperones’ and ‘Translation, ribosomal structure and biogenesis’ (S2 Fig). In the KEGG analysis, the major categories were ‘Translation’, ‘Signal transduction’, ‘Transport and catabolism’, ‘Folding, sorting and degradation’, ‘Endocrine system’, ‘Carbohydrate metabolism’ and ‘Energy metabolism’ (S3 Fig). These annotation and classification will facilitate the following interpretation of the gene function.

Except ribosomal protein, the ten most abundant unigenes were different among tissue types (S2 Table), which reflected the distinct function of each tissue. In hepatopancreas, the typical immune organ of crustacean, *hemocyanin* and *serine proteinase* were abundant as expected, while in the muscle, genes related to muscle formation were highly expressed, such as *skeletal muscle actin 1*, *troponin* and *myosin heavy chain*. It was noted that in the eyestalk, immune related genes, *ferritin*, *metallothionein* (*MT*) and *hemocyanin* were highly expressed, indicating the eyestalk might be involved in the vent crab immune responses as revealed in other decapod species [31–33]. The gill also displayed the character of resistance to stress, with an *anti-lipopolysaccharide factor* (*ALF*) gene and a *heat shock protein* (*HSP*) gene over expressed. ALFs have been reported to have strong antibacterial effect on Gram-positive and Gram-negative bacteria [34,35], and many HSPs are normally up-regulated by heat stimulus, osmotic stress and toxic chemicals, particularly heavy metals [36–39]. These data are important to complete the transcriptomic resources of the vent fauna species, and we will characterize the gene families in detail as follows on different aspects of the crab adaptation to the vent environment.

### Adaptation of visual photopigments and chemical sense

Different marine and fresh water environments require extensive adaptation, and the most affected areas are the acoustic, visual and chemical senses, due to the stimuli of chemical and physical properties of the medium [40]. Understanding the molecular underpinnings of the adaptation may provide broad insight into the crab evolution and behavior.

**Fig 1:**
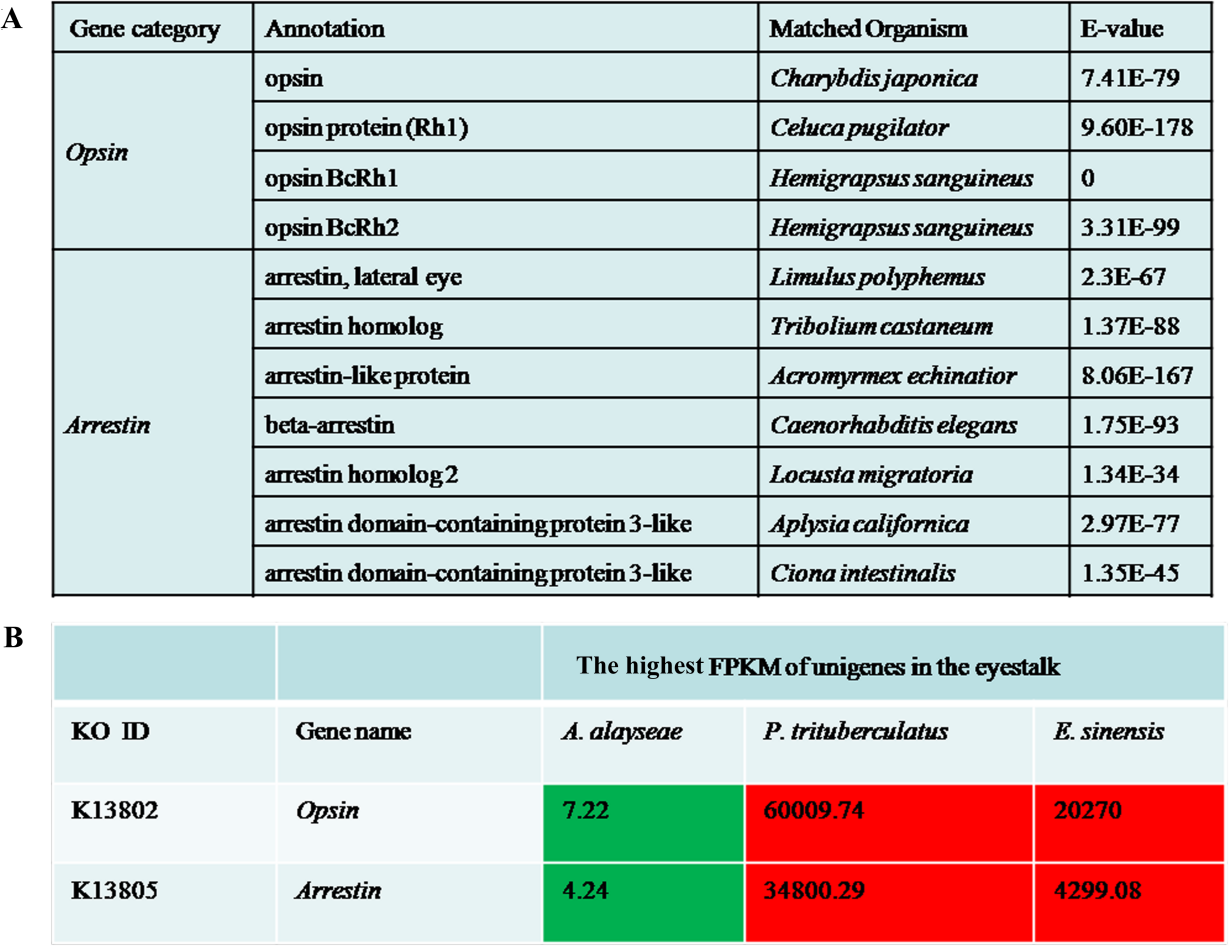
Key phototransduction regulation genes identified in *Austinograea alayseae*. (A) Photoreceptor related genes in the eyestalk of *A*. *alayseae*. (B) Comparison of the highest FPKM values of the phototransduction related genes in the eyestalks of three crabs.

The vent crabs lose eyes and body pigmentation and evolve alternatives in adapting to constant darkness. In the eyestalk transcriptome, all genes involved in the phototransduction-fly pathway, KO04745 (http://www.genome.jp/kegg-bin/show_pathway?ko04745), were found as well as other visual related genes (S3 Table). In addition to a single class of Rhodopsin 1 (Rh1, with a λ_max_ ~500nm) visual pigment, a middle-wavelength-sensitive Rh2 (with λ_max_ 480-530nm) [41] was also identified in *A*. *alayseae* (Fig 1A). However, expression analysis showed that these photoreceptor genes were greatly down regulated in the vent species, as well as their regulators *arrestins*, when compared with that of two shallow water species, *P*. *trituberculatus* and *E*. *sinensis* (Fig 1B). Therefore, it indicates that reduced transcription of phototransduction related genes is consistent with the degenerated retinal function as revealed in the study for cavefish [42].

Since eyes of the vent crabs become vestigial, chemical communication should be the predominant mode of their behavior, such as predation and mating. In the degraded eyestalk, 10 olfactory receptor and three gustatory receptor genes were identified (S3 Table), including four *ionotropic receptors* (*IRs*) and two *metabotropic glutamate receptors*. Thereinto, IR4, IR7, IR8a and IR93a, the insect chemosensory variants of ionotropic glutamate receptors, were reported to be expressed in the eyestalk of crab for the first time. As suggested in the study for the spiny lobsters, *Panulirus argus*, *IRs* expression was detected in not only olfactory tissues, but other types of known chemosensory tissues [43]. The expression of *IRs* in eyestalk of the vent crabs supports the idea that *IRs* may play a more general role in arthropod chemosensation than just mediating detection of olfactory ligands. However, when the transcriptome of two shallow water crab species were searched for sensory related genes, our comparison results showed no difference in the genetic make-up connected to the variable habitats. Studying the important sensory organ, the first pair of antennae, housing their sense of smell, might bring more insights into their sensory function.

### Circadian rhythm regulation and neuropeptide hormones

Many crustaceans are known to exhibit robust circadian rhythms in physiology and behavior, such as feeding, molting, reproduction, hatching and larval release [44,45]. As no light can penetrate through the deep sea, it is interesting to investigate the circadian rhythm of the vent crabs in the deep sea environment.

As a first step to study the molecular mechanisms of biological rhythms in the vent crabs, we mined its transcriptome for homologs of insect proteins known to play roles in the establishment of circadian clock systems (KO04711, http://www.genome.jp/kegg-bin/show_pathway?ko04711). As a result, 13 homologous genes in the insect circadian rhythm pathway were identified (S3 Table), including the core clock proteins, Bmal1, Clock, Period and Timeless, which constitute an interactive feedback loop [46]. Other related proteins were also found, such as casein kinase 1 epsilon, glycogen synthase kinase 3, aryl hydrocarbon receptor nuclear translocator-like protein 1, bZIP transcription factor, and hepatic leukemia factor. Moreover, an *astakine* gene and two *prepro-beta-pigment dispersing hormone* (*PDH*) genes were revealed to be expressed in the eyestalk of *A*. *alayseae*, which have been reported to function in circadian regulation of crustaceans [47–49]. Given the identification of an essentially full complement of circadian transcripts from *A*. *alayseae*, it seems highly likely that these molecules form the basis of a molecular clock in this species.

**Fig 2:**
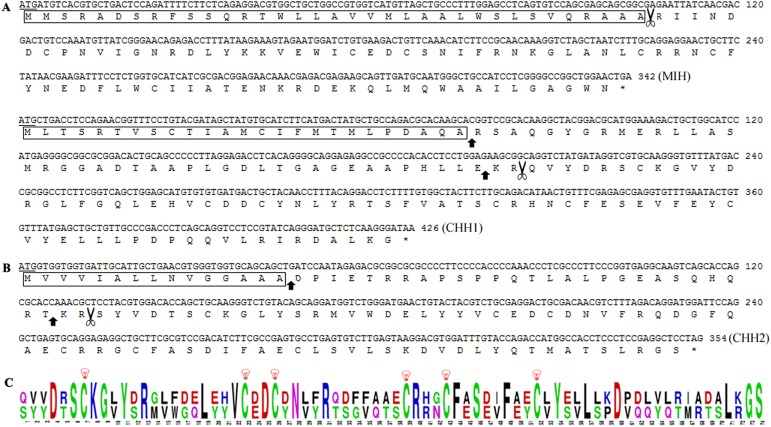
Structure analysis of the three neuropeptides encoding genes in *Austinograea alayseae*. (A) Nucleotide and deduced amino acid sequences of molt-inhibiting hormone (MIH). (B) Nucleotide and deduced amino acid sequences of two crustacean hyperglycemic hormone (CHH) precursors. (C) Web Logos of CHH1 precursor consensus regions compared to CHH2 precursor. The initiation codons are underlined; the signal peptides are marked by boxes; amino acids between vertical arrows indicate CHH precursor related peptide; the cleavage sites are shown by scissors. The six cysteines are marked by bulbs.

Furthermore, the eyestalk synthesizes and secretes several structurally-related peptides belonging to crustacean hyperglycemic hormone (CHH) family, which are considered as major physiological regulators during the crustacean life cycle. Although eyestalks of the hydrothermal vent crabs are degraded, we characterized three transcripts encoding important eyestalk prohormone and hormone of the CHH family in the ‘eyeless’ *A*. *alayseae*, which were one molt-inhibiting hormone (MIH) and two CHH precursors. The deduced hormones contained 141, 117 and 111 amino acids (aa), respectively (Fig 2A and 2B). The MIH included a signal peptide of 35aa and a mature peptide of 78aa (Fig 2A). The CHH1 precursor could be partitioned into a signal peptide of 25aa, followed by a 41aa CPRP (CHH precursor-related peptide), a dibasic cleavage site, and finally a mature peptide of 73aa. CHH2 started with a 15aa signal peptide, followed by a 27aa CPRP and a mature peptide of 73aa (Fig 2B). The mature peptides of the two CHHs were aligned and both have six cysteine residues (Fig 2C), which was a consensus amino acid pattern in the central part of the molecule and predicted to form three disulfide bridges [50]. Thus far, only one CHH has been reported in the hydrothermal vent crabs, Bythograeidae [51]. In this study, CHH2 is a novel neuropeptide found in the vent crabs, while CHH1 is more similar to the reported in *B*. *thermydron*. In *B*. *thermydron*, it starts with a 29aa signal peptide, followed by a 41aa CPRP and a mature peptide of 72aa. Other neuropeptides and related genes were also found in the eyestalk of the crab as shown in S3 Table, including *neurophysin*, *allatostatin*, *buccalin*, *eclosion hormone* and *serotonin receptors*. These genes lay foundation for the study of neuroendocrine regulation in the vent crabs.

### Response to the hypoxia and the reactive oxygen species

In the deep sea, there is devoid of oxygen (O_2_). However, it has been found that the O_2_ consumption rates of hydrothermal-vent animals at low O_2_ environment are similar to those of shallow water species at higher O_2_ environment [52,53], indicating specific adaptation for the O_2_ tension in the former group. Hemocyanins (Hcs) are blue extracellular proteins which are present in high concentrations in the blood of various mollusks and arthropods. Comparable to hemoglobin, Hc is an oxygen-carrier protein. The difference only lies in the nature of the metallic ions that coordinate oxygen, which is iron in case of hemoglobin, whereas copper for hemocyanins. The traditional role of hemocyanin is the transport and storage of molecular oxygen. In this study, six transcripts encoding different hemocyanin subunits were identified (S4 Table). All these transcripts showed high expression in hepatopancreas, which were similar with two shallow water crabs. This reveals that hemocyanin should be a multifunctional protein, and may be potentially involved in the immune responses of decapods with phenoloxidase activity, antimicrobial and antiviral properties as reported in other studies [54–56].

**Fig 3:**
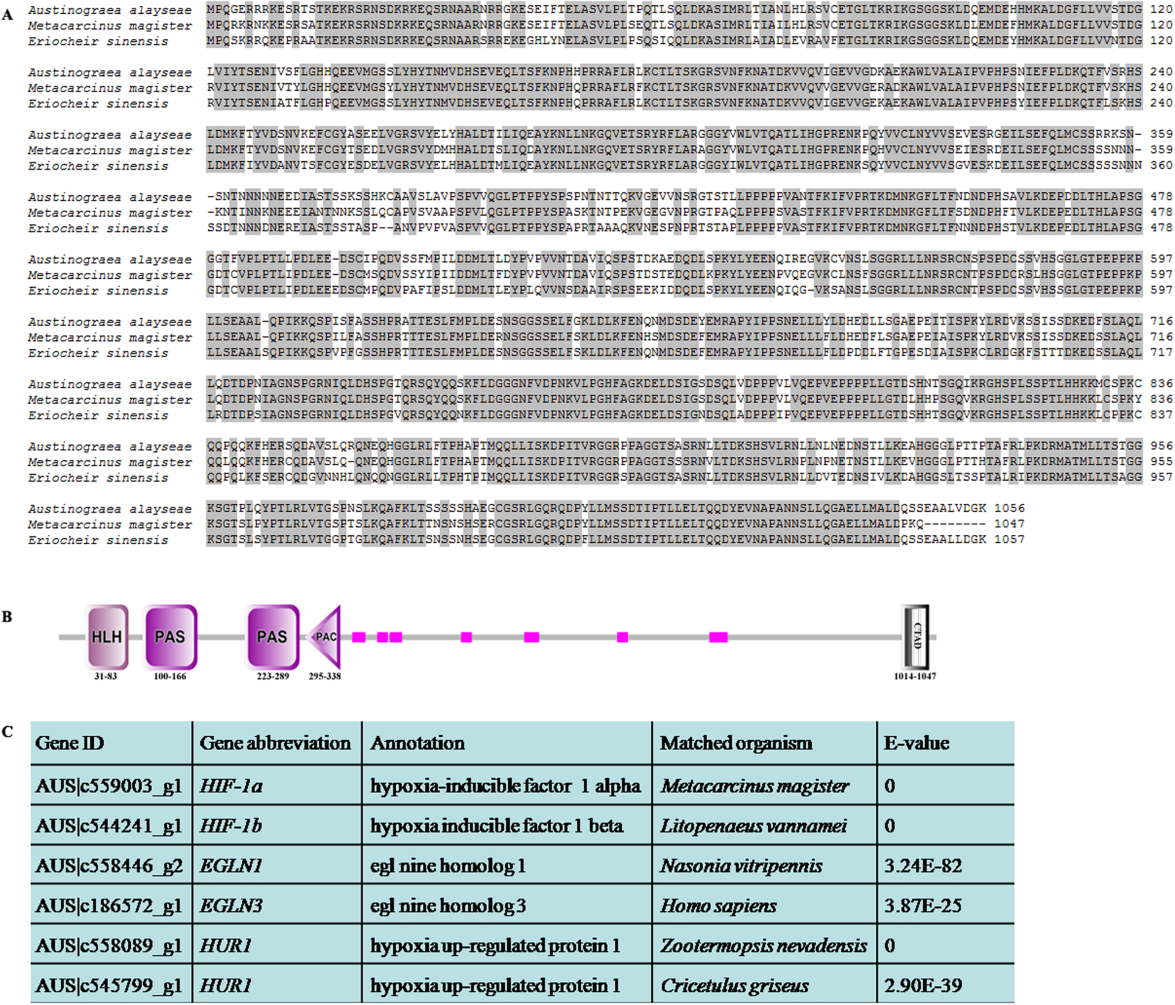
The hypoxia-inducible factor 1 (HIF1) identified in *Austinograea alayseae*. (A) The alignment of HIF-1α from three different crabs. Conserved amino acid residues are in grey. The HIF-1α accession numbers are ABF83561 for *Metacarcinus magister* and AHH85804 for *Eriocheir sinensis*. (B) Specific domains in HIF-1α. HLH: helix loop helix; PAS: PER-ARNT-SIM; PAC: PAS-associated C-terminal; CTAD: transactivating domain. The pink squares represent low complexity regions. (C) HIF1 regulated genes in the transcriptomes.

The transcription factor HIF-1 (hypoxia-inducible factor 1) has been shown to play an essential role in maintenance of homeostatic responses to hypoxia [57]. It is a dimeric protein consisting of a HIF-1α and a HIF-1ß subunits. HIF-1ß (also known as the aryl hydrocarbon receptor nuclear translocator, ARNT protein) is a common subunit for several transcription factors and constitutively expressed, while the HIF-1α subunit is unique to HIF-1 and O_2_-regulation [58]. From the vent crab transcriptomes, genes encoding HIF-1ɑ and HIF-1ß were both identified (Fig 3). The HIF-1α was composed of 1,056aa (Fig 3A), containing a typical HLH (helix loop helix) domain, two distinct PAS (PER-ARNT-SIM) domains, a PAC (PAS-associated C-terminal) domain and a transactivating domain (CTAD) (Fig 3B). Compared with those of other crabs, the HIF-1ɑ sequence of the vent crab showed high similarity with *Metacarcinus magister* and *E*. *sinensis*, with an identity of 88% and 83%, respectively (Fig 3A). Moreover, an oxygen sensor, Egl nine homolog 1 (EGLN1), also known as prolyl hydroxylase domain 2 (PHD2) protein, was found in the transcriptomes (Fig 3C), which have been reported to play pivotal roles in the HIF-1α pathway regulation [59]. Egl nine homolog 3 (EGLN3) was also detected, while its role in the regulation of HIF-1α has not been established. Moreover, two hypoxia up-regulated proteins were discovered in the transcriptomes (Fig 3C), which might be involved in the hypoxia response of the crab.

The excessive production of reactive oxygen species (ROS) is another impact factor for the vent species, which is resulted from increased oxidative stress and detrimental to the cell [60]. Metals are known to enhance the production of ROS, and consequently increase the oxyradical stress for the species [61]. Many oxidase related genes existed in the vent crab transcriptomes, including *catalase*, *dual oxidase*, *glutathione peroxidase* (*GPx*), *selenoprotein W* and *superoxide dismutase* (*SOD*) (S4 Table). Three families of SODs were found and copper/zinc superoxide dismutase and manganese superoxide dismutase predominated.

The thioredoxin (Trx) is the main ubiquitously expressed thiol-reducing antioxidant systems [62]. In the transcriptomes, many thioredoxin-like proteins, Trx domain-containing proteins and Trx reductases, were found (S4 Table). Thereinto, two forms of Trx were identified, Trx1 and Trx2, both with conserved catalytic center -Cys-Gly-Pro-Cys- (-CGPC-). Trx1 exists primarily in cytosol and is also found in the nucleus and blood plasma [63], whereas Trx2 is a mitochondrial form with an N-terminal mitochondrial translocation and signal peptide, and lacks the other cysteine residues that are found in Trx1 [64]. It is noted that Trx2 of the *A*. *alayseae* showed high identity with that of crab *P*. *trituberculatus*, while Trx1 in the vent crabs was more similar with that of a sponge *Amphimedon queenslandica*. Therefore, Trx1 in *A*. *alayseae* might be a new form of Trx in the crabs.

### Ion transport and metal detoxification

Extremely high hydrostatic pressure is one of the major characteristics of the hydrothermal vent. Gill is one of the most important organs involved in osmoregulation in crabs. Two crucial enzymes responsible for ion transport in crustacean gills, Na^+^/K^+^-ATPase, V-type H^+^ -ATPase, were both identified with high expression level in the gill transcriptome (S5 Table). Other regulators were also found, including solute carrier (SLC) family members, sodium/hydrogen exchanger (SLC9) and urea transporter 2 (SLC14). Notably, many SLC members related to metal ion transport were expressed in the gill, including SLC11 (3 unigenes), SLC30 (6 unigenes), SLC31 (2 unigenes), SLC39 (11 unigenes). Among them, SLC11 members are proton-coupled transporters involved in Fe^2+^, Cd^2+^, Co^2+^, Cu^1+^, Mn^2+^, Ni^2+^, Pb^2+^, Zn^2+^ transport. SLC30 and SLC39 are zinc transporters, while SLC31 are responsible for the copper transport. These gene encoding proteins are expected to facilitate the heavy metal transport of the crabs in the vent environment.

**Fig 4:**
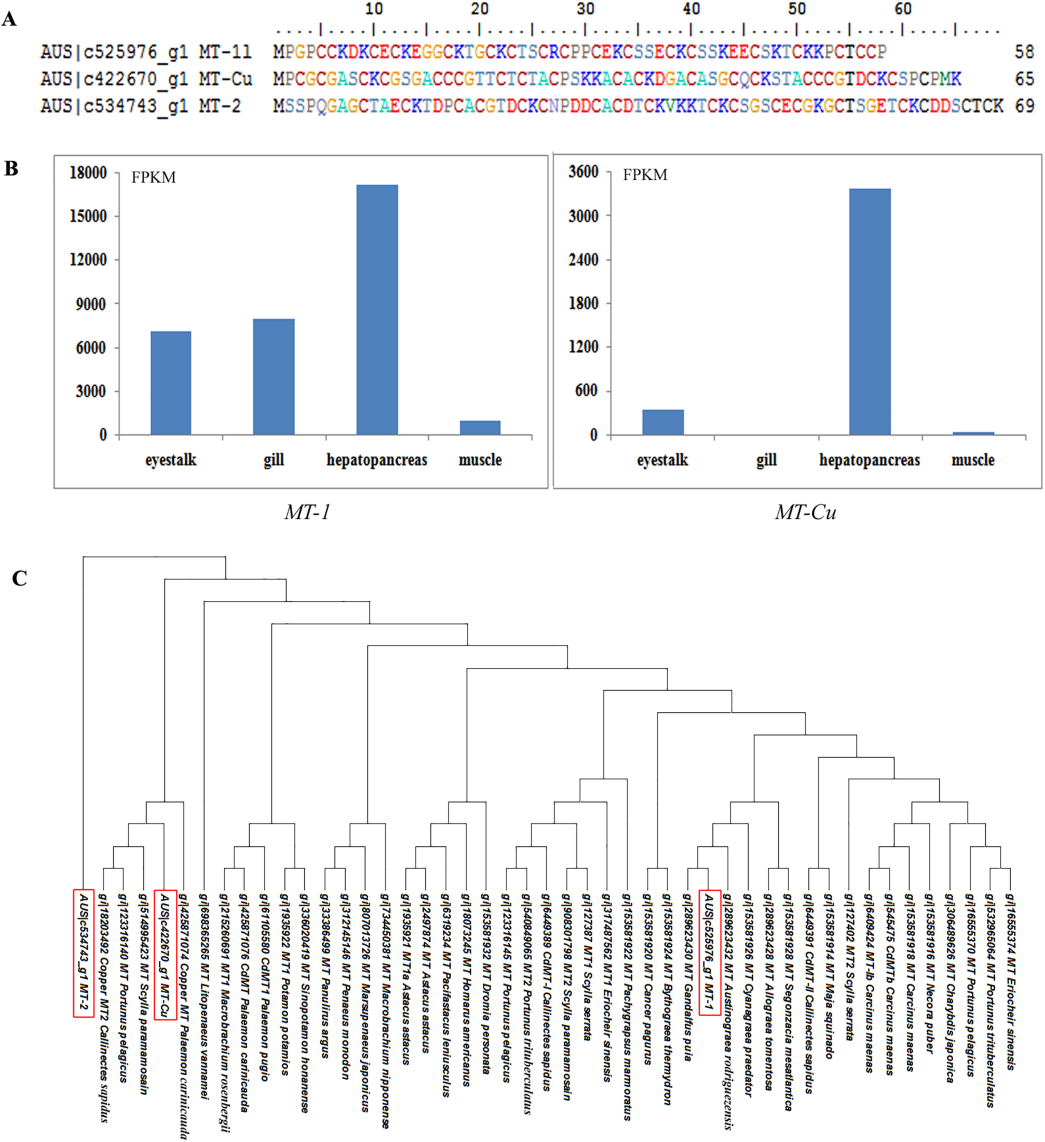
The metallothioneins (MTs) identified in *Austinograea alayseae*. (A) The alignment of the three MTs of *A*. *alayseae*. (B) Comparison of the FPKM values of two *MT* genes in four tissues. (C) Phylogenetic tree of the MTs from decapods. The MTs in *A*. *alayseae* are marked by red boxes.

High levels of hydrogen sulfide and heavy metals are the typical feature of the hydrothermal vent, which requires specific adaptation for this condition. Metallothioneins (MTs) are unusual low molecular weight intracellular proteins found in a wide variety of vertebrates and invertebrates, displaying heat stability, high cysteine content and nonenzymatic nature. MTs can act as a metal reservoir to maintain the homeostasis of essential metals, as well as a main effector in the detoxification of excessive metals [65]. Three different MT cDNA forms were isolated from *A*. *alayseae* transcriptome, named MT-1, MT-Cu, MT-2. The open reading frames were 58aa, 65aa and 69aa in length, respectively (Fig 4A), of which 18aa, 21aa and 18aa were cysteine residues. By protein BLAST, MT-1 showed high similarity (98%) with another vent crab, *G*. *puia*. MT-Cu was annotated as copper-specific metallothionein-2 in *Callinectes sapidus* with identity 78%, while MT-2 was 97% identical to an abalone, *Haliotis discus hannai*. As shown in the phylogenetic tree for MTs of decapods, MT-1 of *A*. *alayseae* was clustered with the hydrothermal vent crab family group, Bythograeidae, while the other two distributed in separated clusters (Fig 4C). Therefore, MT-Cu and MT-2 might be novel isoforms identified in hydrothermal vent crabs. Among them, *MT-1* showed high expression in all four tissues tested, while *MT-2* was lowly expressed in four tissues (Fig 4B). Notably, the expression of *MT-Cu* was extremely high in hepatopancreas, a vital immune organ. It has been reported that hydrothermal vent organisms are exposed to copper concentrations up to a thousand times higher than those in the oceanic water [66], which might result in the high expression of copper specific MT.

### Positively selected genes and immune adaptation

**Fig 5:**
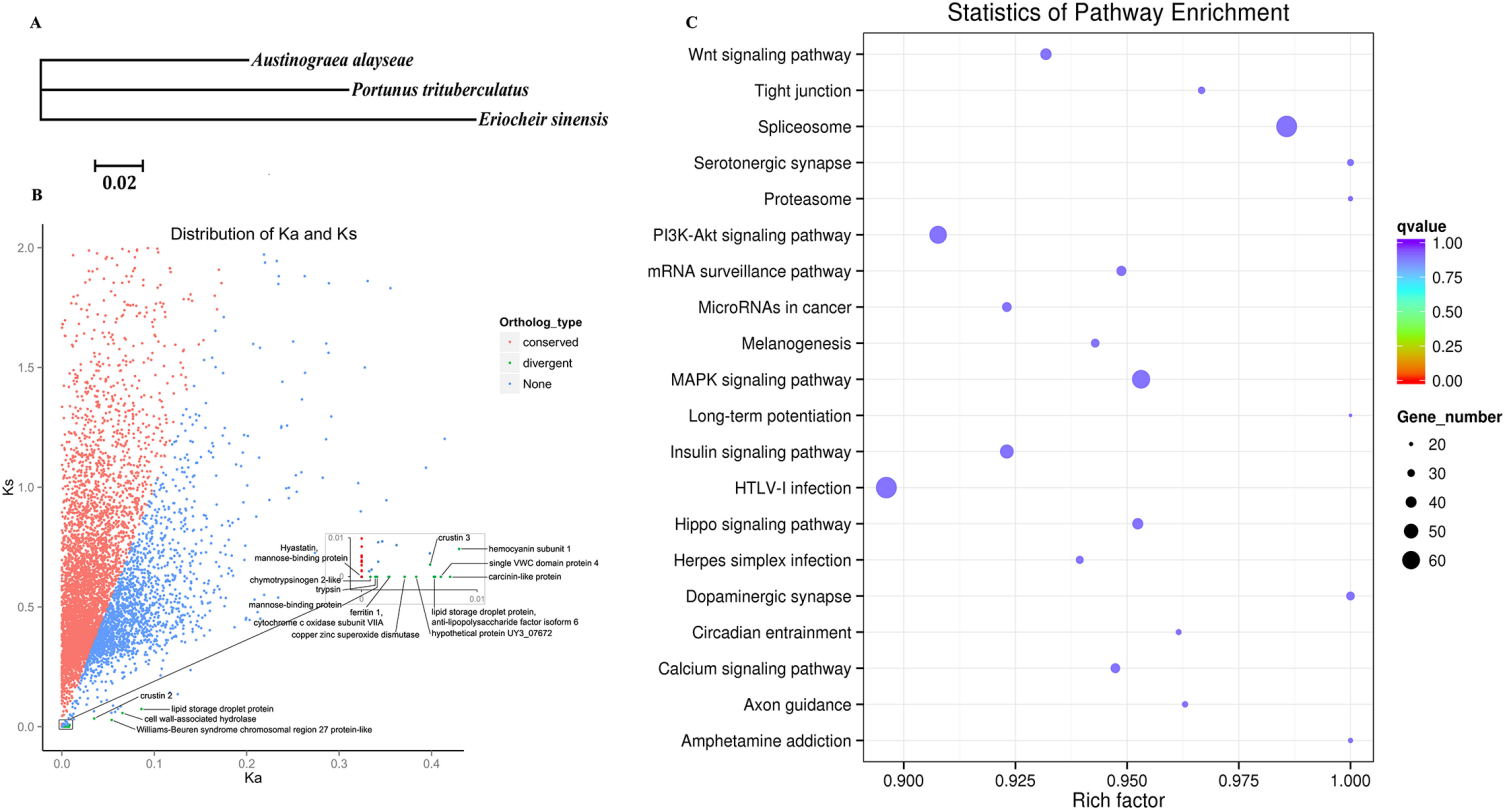
Orthologous genes analysis results. (A) Phylogenetic tree of five arthropods based on the orthologous genes. (B) Distribution of Ka/Ks for the orthologous genes among three crabs. We define genes with Ka/Ks >1 as divergent genes, and genes with Ka/Ks < 0.01 as conserved genes. (C) KEGG analysis for the conserved genes.

Among three crabs, *A*. *alayseae*, *P*. *trituberculatus* and *E*. *sinensis*, 6,932 orthologous genes were identified, while among five arthropods including three crabs, *Daphnia pulex* and *Drosophilla virilis*, 1,145 orthologous genes were identified and the phylogenic tree was constructed correspondingly (Fig 5A). Among them, *A*. *alayseae* was closest to *P*. *trituberculatus*, which is the representation of sea crab. The sea crab was then clustered with *E*. *sinensis*, which is a catadromous species with most of its life spending in freshwater and larvae in salt water. This phylogenetic relationship is consistent with the previous study [67].

Ka, Ks and Ka/Ks ratios were calculated for the 6,932 orthologous genes of three crabs, resulting in 19 genes with Ka/Ks >1 and 5,040 genes with Ka/Ks < 0.1 (Fig 5B). Among the conserved genes, Wnt, MAPK, PI3K-Akt, insulin signaling pathway and hippo signaling pathway were the dominant categories based on KEGG enrichment analysis (Fig 5C). These are all related to development, indicating a conserved way of growth and development in crabs. In the 19 divergent genes (Fig 5B and Table 2), most of them were involved in immune responses. For instance, three *crustin* genes were identified as *crustin2*, *crustin3* and *carcinin-like protein* genes. *Crustin* is an antibacterial peptide family composed of three members *custin1-3*, with a characteristic four-disulphide core-containing whey acidic protein (WAP) domain and a broad antibacterial spectrum [68]. Two other antibacterial peptides, hyastatin and anti-lipopolysaccharide factor isoform 6 were also discovered. Moreover, two mannose-binding proteins (MBLs) were positively selected, which are members of mannose pathway of complement system and involved in crab adaptive immunity. Two genes involved in the antioxidant system were also found, including *ferritin 1* and *copper zinc superoxide dismutase* (*SOD1*), while the identified *single VWC domain protein 4* (*SVC4*) can respond to environmental challenges, such as bacterial infection and nutritional status [69]. The discovered *Dscam* gene plays an essential function in neuronal wiring and appears to be involved in innate immune responses in many arthropods [70]. In addition, two serine-type endopeptidase chymotrypsinogen 2-like (Ctrbl) and trypsin, associated with proteolysis and metabolism, were also revealed to be under selection. These genes provide nice candidates to study their function in the adaptive evolution of crab species.

**Table 2:** Function categories of positively selected genes in three crabs, *Austinograea alayseaee*, *Portunus trituberculutus* and *Eriocheir sinensis*.

Orthology ID	Gene name	Annotation	Function
OG06005	COX7A	cytochrome c oxidase subunit VIIA putative	Energy metabolism
OG09209	WHL	cell wall-associated hydrolase	Metabolism
OG12001	Hyastatin	antimicrobial peptide hyastatin	Immunity
OG12033	Ctrb1	chymotrypsinogen 2-like	Proteolysis
OG12037	MBL	mannose-binding protein	Immunity
OG12105	ferritin 1	ferritin 1	Immunity
OG12337		hypothetical protein UY3_07672 [Chelonia mydas]	Unknown
OG12436	crustin3	crustin 3	Immunity
OG12521	trypsin	trypsin	Proteolysis
OG12637	crustin2	crustin 2	Immunity
OG13252	ALF6	anti-lipopolysaccharide factor isoform 6	Immunity
OG13368	carcinin-like	carcinin-like protein	Immunity
OG13448	SOD1	copper zinc superoxide dismutase	Immunity
OG13939	Hc1	hemocyanin subunit 1	Energy metabolism
OG14016	WBSCR27	Williams-Beuren syndrome chromosomal region 27 protein-like	Unknown
OG14400	MBL	mannose-binding protein	Immunity
OG14461	SVC	single VWC domain protein 4	Immunity
OG14472	Dscam2	DSCAM	Immunity
OG14474	LSDP	lipid storage droplet protein	Metabolism

Considering the significance of immune genes in the evolution of the crabs, we characterized 33 additional immune genes by searching the transcriptome of hepatopancreas, especially focus on genes related to pathogenic microorganism stress of the vent (S4 Table). There were 12 antimicrobial peptides, including five ALFs, seven crustins, and 21 genes involved in prophenoloxidase-activating system (serine protease, serine proteinase inhibitor, pacifastin, prophenoloxidase and serpin). Exploring these genes will contribute to understand the mechanisms of pathogens elimination and the symbiont phenomenon in the hydrothermal vent crabs.

## Conclusions

The transcriptome analysis of *A*. *alayseae* revealed a comprehensive set of genes expressed in four different tissues, resulting in 725,461 unigenes and 134,489 annotated genes. Candidate genes related to harsh condition adaptation were identified, such as genes related to hypoxia, metal detoxification, high osmotic pressure and pathogens. Sequences and structures of many candidate genes were characterized, including *MIH*, *CHHs*, *HIF1* and *MTs*. Orthologs among three crabs revealed 19 PSGs and most of them were involved in immune responses, indicating their important roles in the environment adaptation in crabs. This first transcriptome of the hydrothermal crabs sets the stage for expanding the genetic resources available for vent species, and provides candidate genes that are likely tied with physiological adaptation to the extreme environment.

## Supporting information

S1 Table Unigene annotation in *Austinograea alayseae*. (RAR)

S2 Table The ten most abundant unigenes in each tissue of *Austinograea alayseae*. (DOCX)

S3 Table Sensory, circadian rhythm, neuropeptide and hormone related genes in the eyestalk transcriptome of *Austinograea alayseae*. (DOCX)

S4 Table Key genes associated with stress adaptation for the hydrothermal vent environment in transcriptomes of *Austinograea alayseae*. (DOCX)

S5 Table Osmoregulation related genes and metal transport solute carrier (SLC) family members in the gill transcriptome of *Austinograea alayseae*. (DOCX)

S1 Fig GO distribution of all unigenes in the transcriptomes of *Austinograea alayseae*. (TIF)

S2 Fig KOG function classification of all unigenes in the transcriptomes of *Austinograea alayseae*. (TIF)

S3 Fig KEGG classification of all unigenes in the transcriptomes of *Austinograea alayseae*. A: Cellular Processes; B: Environmental Information Processing; C: Genetic Information Processing; D: Metabolism; E: Organismal Systems.
(TIF)
