# Supplementary material for: Exploring the molecular basis of adaptive evolution in hydrothermal vent crab Austinograea alayseae by transcriptome analysis
Source: PLoS One. 2017 May 26;12(5):e0178417. doi: 10.1371/journal.pone.0178417 (PMC5446156; doi:10.1371/journal.pone.0178417)
Supplement: S2 Table — (DOCX) [file pone.0178417.s002.docx]

**S2 Table. The ten most abundant unigenes in each tissue of *Austinograea alayseae*.**

| Rank of expression | Eyestalk | Gill | Hepatopancreas | Muscle |
| --- | --- | --- | --- | --- |
| 1 | ubiquitin | ubiquitin | ferritin | muscle LIM protein |
| 2 | ferritin | epididymal secretory glutathione peroxidase | hemocyanin subunit 6 | skeletal muscle actin 1 |
| 3 | metallothionein | ferritin | hemocyanin subunit 4 | ubiquitin |
| 4 | hemocyanin subunit 6 | anti-lipopolysaccharide factor isoform 3 | ubiquitin | troponin I |
| 5 | skeletal muscle actin 1 | arginine kinase | trypsin-like serine proteinase 4 | troponin T |
| 6 | beta-actin | cystatin A | chymotrypsin-like proteinase | troponin C |
| 7 | translationally controlled tumor protein | glucose dehydrogenase | hemocyanin subunit 1 | myosin heavy chain type b |
| 8 | alpha-tubulin | alpha-tubulin | hemocyanin subunit 3 | allergen tropomyosin |
| 9 | arginine kinase | heat shock protein 70 | zinc proteinase Mpc1 | crustin 3 |
| 10 | histone H4 | translationally controlled tumor protein | peritrophin | arginine kinase |
