# Supplementary material for: Exploring the molecular basis of adaptive evolution in hydrothermal vent crab Austinograea alayseae by transcriptome analysis
Source: PLoS One. 2017 May 26;12(5):e0178417. doi: 10.1371/journal.pone.0178417 (PMC5446156; doi:10.1371/journal.pone.0178417)
Supplement: S3 Table — (DOCX) [file pone.0178417.s003.docx]

**S3 Table. Sensory, circadian rhythm, neuropeptide and hormone related gene in the eyestalk transcriptome of *Austinograea alayseae*.**

| **Unigene ID** | **Annotation** | **Matched Organism** | **E-value** |
| --- | --- | --- | --- |
| **Visual sensory** | | | |
| AUS\|c527513_g2 | eyegone isoform X1 | *Tribolium castaneum* | 1.34E-38 |
| AUS\|c561092_g1 | eya | *Euprymna scolopes* | 4.17E-136 |
| AUS\|c551806_g1 | cat eye syndrome critical region protein 5 | *Zootermopsis nevadensis* | 5.88E-151 |
| AUS\|c554105_g1 | popeye domain-containing protein 3 | *Gallus gallus* | 7.32E-60 |
| AUS\|c553175_g5 | popeye domain-containing protein 2 | *Homo sapiens* | 2.62E-20 |
| AUS\|c557930_g1 | eye-specific diacylglycerol kinase | *Drosophila melanogaster* | 0 |
| **Olfactory sensory** | | | |
| AUS\|c493575_g1 | otoferlin | *Acromyrmex echinatior* | 4.66E-45 |
| AUS\|c548310_g2 | olfactory ionotropic receptor IR4 | *Panulirus argus* | 5.21E-55 |
| AUS\|c450320_g1 | olfactory ionotropic receptor IR7 | *Panulirus argus* | 2.21E-19 |
| AUS\|c558039_g3 | olfactory ionotropic receptor IR8a | *Panulirus argus* | 1.14E-121 |
| AUS\|c539655_g2 | olfactory ionotropic receptor IR93a | *Panulirus argus* | 0 |
| AUS\|c557809_g1 | olfactory receptor 11G2-like | *Equus caballus* | 7.55E-07 |
| AUS\|c558008_g1 | substance-K receptor-like | *Metaseiulus occidentalis* | 5.83E-58 |
| AUS\|c553874_g1 | protein lunapark-B isoform X2 | *Nasonia vitripennis* | 4.72E-34 |
| AUS\|c545216_g1 | protein JTB | *Myotis brandtii* | 667E-06 |
| AUS\|c562368_g4 | colmedin | *Culex quinquefasciatus* | 0 |
| **Gustatory sensory** | | | |
| AUS\|c154576_g1 | tyramine/octopamine receptor | *Macrobrachium rosenbergii* | 3.88E-59 |
| AUS\|c528298_g1 | metabotropic glutamate receptor 3 | *Stegodyphus mimosarum* | 3.53E-19 |
| AUS\|c563049_g1 | metabotropic glutamate receptor | *Drosophila melanogaster* | 1.87E-68 |
| **Circadian rhythm** | | | |
| AUS\|c556317_g2 | bmal1a | *Pacifastacus leniusculus* | 0 |
| AUS\|c561611_g1 | cryptochrome circadian clock 1 (CRY1) | *Elephantulus edwardii* | 2.81E-53 |
| AUS\|c563072_g1 | period | *Eurydice pulchra* | 0 |
| AUS\|c561289_g2 | timeless-like protein | *Camponotus floridanus* | 3.7E-29 |
| AUS\|c563318_g1 | timeless | *Eurydice pulchra* | 6.6E-97 |
| AUS\|c504278_g1 | casein kinase 1 epsilon | *Eurydice pulchra* | 1.59E-179 |
| AUS\|c547746_g1 | glycogen synthase kinase 3 alpha | *Sarcophilus harrisii* | 1.21E-124 |
| AUS\|c751137_g1 | glycogen synthase kinase 3 beta | *Stegodyphus mimosarum* | 3.47E-36 |
| AUS\|c526507_g1 | aryl hydrocarbon receptor nuclear translocator-like protein 1 | *Tyto alba* | 1.19E-10 |
| AUS\|c546136_g3 | bZIP transcription factor | *Aphaenogaster texana* | 4.37E-46 |
| AUS\|c529025_g1 | hepatic leukemia factor-like | *Astyanax mexicanus* | 1.91E-23 |
| AUS\|c529025_g2 | hepatic leukemia factor | *Mus musculus* | 1.27E-10 |
| AUS\|c525890_g2 | hepatic leukemia factor | *Mus musculus* | 3.09E-20 |
| AUS\|c543149_g1 | melatonin receptor type 1C | *Xenopus laevis* | 1.48E-09 |
| AUS\|c527710_g1 | astakine | *Eriocheir sinensis* | 1.44E-27 |
| AUS\|c491532_g1 | prepro-beta-pigment dispersing hormone I | *Cancer productus* | 1.47E-131 |
| AUS\|c497950_g1 | prepro-beta-pigment dispersing hormone IIa | *Cancer productus* | 2.12E-66 |
| **Neuropeptide and Hormone** | | | |
| AUS\|c551829_g1 | molt-inhibiting hormone | *Cancer pagurus* | 4E-44 |
| AUS\|c550346_g1 | prepro crustacean hyperglycemic hormone pericardial organ isoform, CHH1 | *Potamon ibericum* | 5E-63 |
| AUS\|c551228_g1 | CHH2 | *Scylla paramamosain* | 1E-30 |
| AUS\|c527988_g1 | vasotocin-neurophysin | *Scylla paramamosain* | 2E-57 |
| AUS\|c487962_g1 | allatostatin precursor protein | *Panulirus interruptus* | 6E-38 |
| AUS\|c553771_g1 | buccalin precursor | *Aplysia californica* | 1.42E-08 |
| AUS\|c559031_g1 | eclosion hormone | *Scylla paramamosain* | 1E-24 |
| AUS\|c243084_g1 | type 2 serotonin receptor | *Panulirus interruptus* | 9.53E-36 |
| AUS\|c557467_g1 | type 1 serotonin receptor | *Procambarus clarkii* | 0 |
