# Supplementary material for: Exploring the molecular basis of adaptive evolution in hydrothermal vent crab Austinograea alayseae by transcriptome analysis
Source: PLoS One. 2017 May 26;12(5):e0178417. doi: 10.1371/journal.pone.0178417 (PMC5446156; doi:10.1371/journal.pone.0178417)
Supplement: S4 Table — (DOCX) [file pone.0178417.s004.docx]

**S4 Table. Key genes associated with stress adaptation for the hydrothermal vent environment in transcriptomes of *Austinograea alayseae*.**

| **Gene category** | **Unigene ID** | **Annotation** | **Matched Organism** | **E-value** |
| --- | --- | --- | --- | --- |
| **Hypoxia and Oxidative stress** | | | | |
| *Hemocyanin* | AUS\|c562859_g1 | hemocyanin | *Portunus pelagicus* | 5.93E-81 |
|  | AUS\|c562824_g1 | hemocyanin subunit | *Callinectes sapidus* | 1.01E-31 |
|  | AUS\|c558281_g2 | hemocyanin subunit 1 | *Metacarcinus magister* | 1.03E-148 |
|  | AUS\|c357597_g1 | hemocyanin 2 | *Pacifastacus leniusculus* | 6.22E-109 |
|  | AUS\|c516811_g1 | hemocyanin subunit 3 | *Metacarcinus magister* | 5.48E-66 |
|  | AUS\|c562859_g3 | hemocyanin subunit 6 | *Eriocheir sinensis* | 0 |
| *Catalase* | AUS\|c745874_g1 | catalase | *Haliotis discus discus* | 1.83E-103 |
|  | AUS\|c653336_g1 | catalase | *Haliotis discus discus* | 1.19E-40 |
| *Dual oxidase* | AUS\|c559068_g1 | dual oxidase 2 | *Zootermopsis nevadensis* | 0 |
|  | AUS\|c807569_g1 | dual oxidase 2-like | *Strongylocentrotus purpuratus* | 1.27E-08 |
| *GPx* | AUS\|c533274_g1 | glutathione peroxidase | *Metapenaeus ensis* |  |
|  | AUS\|c529506_g1 | glutathione peroxidase | *Procambarus clarkii* |  |
|  | AUS\|c543665_g1 | selenium-dependent glutathione peroxidase | *Scylla paramamosain* |  |
|  | AUS\|c523008_g1 | phospholipid glutathione peroxidase | *Macrophthalmus japonicas* |  |
| *SOD* | AUS\|c63709_g1 | superoxide dismutase, putative | *Ichthyophthirius multifiliis* | 4.55E-62 |
|  | AUS\|c574623_g1 | superoxide dismutase | Aplysia californica | 3.84E-50 |
|  | AUS\|c622390_g1 | extracellular copper-zinc superoxide dismutase | *Portunus trituberculatus* | 4.50E-57 |
|  | AUS\|c93835_g1 | copper/zinc superoxide dismutase CuZnSODb | *Anemonia viridis* | 7.70E-37 |
|  | AUS\|c89729_g1 | copper/zinc superoxide dismutase | *Eriocheir sinensis* | 2.97E-27 |
|  | AUS\|c809777_g1 | superoxide dismutase [Cu-Zn], partial | *Stegodyphus mimosarum* | 7.31E-41 |
|  | AUS\|c551942_g1 | copper zinc superoxide dismutase | *Callinectes sapidus* | 4.29E-81 |
|  | AUS\|c546265_g1 | copper/zinc superoxide dismutase isoform 2 | *Marsupenaeus japonicus* | 2.08E-66 |
|  | AUS\|c791847_g1 | Cu-Zn superoxide dismutase | *Apostichopus japonicus* | 4.51E-35 |
|  | AUS\|c535132_g1 | extracellular Cu, Zn-superoxide dismutase | *Callinectes sapidus* | 8.61E-64 |
|  | AUS\|c594053_g1 | copper zinc superoxide dismutase | *Callinectes sapidus* | 7.14E-56 |
|  | AUS\|c786861_g1 | superoxide dismutase [Mn], mitochondrial-like | *Amphimedon queenslandica* | 5.88E-40 |
|  | AUS\|c679503_g1 | superoxide dismutase [Mn], mitochondrial | *Calypte anna* | 2.21E-34 |
|  | AUS\|c577856_g1 | Mn-superoxide dismutase | *Haliotis rufescens* | 3.00E-45 |
|  | AUS\|c755058_g1 | manganese superoxide dismutase | *Chironomus riparius* | 3.11E-38 |
|  | AUS\|c679719_g1 | manganese superoxide dismutase | *Hypophthalmichthys molitrix* | 1.33E-30 |
|  | AUS\|c543594_g2 | cytoplasmic manganese superoxide dismutase | *Segonzacia mesatlantica* | 0 |
|  | AUS\|c59753_g1 | superoxide dismutase, Fe-Mn family | *Strigomonas culicis* | 8.47E-43 |
|  | AUS\|c17187_g1 | superoxide dismutase [Fe]-like | *Ceratitis capitata* | 2.14E-76 |
| *Selenoprotein W* | AUS\|c676372_g1 | selenoprotein W | *Astyanax mexicanus* | 8.25E-13 |
|  | AUS\|c672387_g1 | selenoprotein W-like | *Python bivittatus* | 2.41E-08 |
|  | AUS\|c800914_g1 | selenoprotein W-like | *Python bivittatus* | 2.17E-11 |
|  | AUaS\|c586830_g1 | selenoprotein W2 | *Artemia franciscana* | 4.94E-17 |
|  | AUS\|c105444_g1 | selenoprotein W2 | *Artemia franciscana* | 1.99E-08 |
| *Thioredoxin* | AUS\|c210857_g1 | thioredoxin-like, thioredoxin 1 | *Amphimedon queenslandica* | 3.71E-26 |
|  | AUS\|c517941_g1 | thioredoxin 2 | *Portunus trituberculatus* | 1.02E-67 |
| **Chemical stress** | | | | |
| *Metallothionein* | AUS\|c525976_g1 | mt gene for metallothionein (MT-1) | *Cyanagraea praedator* | 9.14E-170 |
|  | AUS\|c422670_g1 | copper-specific metallothionein-2 (MT-Cu) | *Callinectes sapidus* | 8.10E-12 |
|  | AUS\|c534743_g1 | metallothionein (MT-2) | *Haliotis diversicolor supertexta* | 6.35E-06 |
| *Ferritin* | AUS\|c31576_g1 | ferritin 1 | *Eriocheir sinensis* | 2.56E-54 |
|  | AUS\|c557662_g1 | ferritin 2 | *Eriocheir sinensis* | 3.72E-94 |
|  | AUS\|c509576_g1 | ferritin | *Haliotis discus hannai* | 4.76E-123 |
|  | AUS\|c44954_g1 | ferritin | *Haliotis rufescens* | 8.12E-123 |
| **Pathogenic microorganism stress** | | | | |
| *Prophenoloxidase-activating system* | | | | |
| *Serine protease* | AUS\|c554340_g1 | serine protease | *Fenneropenaeus chinensis* | 2.54E-22 |
|  | AUS\|c543493_g1 | serine protease | *Scylla paramamosain* | 4.11E-63 |
|  | AUS\|c428045_g1 | serine protease | *Scylla paramamosain* | 1.51E-33 |
|  | AUS\|c517161_g1 | serine protease | *Penaeus monodon* | 8.11E-39 |
|  | AUS\|c515713_g1 | serine protease like protein | *Cephonodes hylas* | 3.53E-13 |
|  | AUS\|c532579_g1 | serine protease 1 | *Litopenaeus vannamei* | 6.99E-119 |
|  | AUS\|c202394_g1 | serine protease H51 | *Tribolium castaneum* | 9.70E-13 |
|  | AUS\|c470504_g1 | serine proteinase inhibitor | *Pacifastacus leniusculus* | 6.71E-17 |
|  | AUS\|c795406_g1 | Kazal-type serine protease inhibitor | *Pinctada fucata* | 3.07E-06 |
|  | AUS\|c520773_g1 | Kazal-like serine protease inhibitor-like protein | *Scylla paramamosain* | 3.33E-09 |
| *Pacifastin* | AUS\|c747984_g1 | pacifastin heavy chain precursor | *Scylla paramamosain* | 9.01E-40 |
|  | AUS\|c744585_g1 | pacifastin heavy chain precursor | *Pacifastacus leniusculus* | 1.84E-123 |
|  | AUS\|c610778_g1 | pacifastin heavy chain | *Macrobrachium rosenbergii* | 3.74E-46 |
|  | AUS\|c562054_g1 | pacifastin-related serine protease inhibitor | *Portunus trituberculatus* | 1.36E-129 |
|  | AUS\|c533678_g2 | pacifastin-related serine protease inhibitor | *Portunus trituberculatus* | 6.46E-06 |
| *Prophenoloxidase* | AUS\|c551199_g1 | prophenoloxidase | *Cancer pagurus* | 0 |
|  | AUS\|c86352_g1 | prophenoloxidase-activating factor | *Eriocheir sinensis* | 2.91E-76 |
|  | AUS\|c553174_g3 | prophenoloxidase activating enzyme III | *Callinectes sapidus* | 4.73E-165 |
|  | AUS\|c68584_g1 | prophenoloxidase activating factor serine proteinase | *Scylla serrata* | 3.68E-59 |
|  | AUS\|c717233_g1 | phenoloxidase activating factor | *Portunus trituberculatus* | 2.43E-29 |
| *Serpin* | AUS\|c409015_g1 | serpin B13 | *Ornithorhynchus anatinus* | 8.02E-09 |
| *Antimicrobial peptides* | | | | |
| *ALF* | AUS\|c570416_g1 | anti-lipopolysaccharide factor 3 | *Eriocheir sinensis* | 2.23E-56 |
|  | AUS\|c470637_g1 | anti-lipopolysaccharide factor | *Eriocheir sinensis* | 2.42E-70 |
|  | AUS\|c454934_g1 | anti-lipopolysaccharide factor | *Portunus trituberculatus* | 1.67 E-8 |
|  | AUS\|c41256_g1 | anti-lipopolysaccharide factor isoform 3 | *Portunus trituberculatus* | 4.98E-33 |
|  | AUS\|c117300_g1 | anti-lipopolysaccharide factor isoform 6 | *Portunus trituberculatus* | 1.19E-64 |
| *Crustin* | AUS\|c718485_g1 | crustin | *Scylla tranquebarica* | 5.9E-34 |
|  | AUS\|c541271_g2 | crustin 3 | *Panulirus japonicus* | 6.27E-16 |
|  | AUS\|c535665_g1 | crustin-2 | *Eriocheir sinensis* | 6.93E-33 |
|  | AUS\|c521157_g1 | crustin 2 | *Portunus trituberculatus* | 7.83E-14 |
|  | AUS\|c497789_g1 | crustin 3 | *Portunus trituberculatus* | 4.65E-23 |
|  | AUS\|c433370_g1 | crustin-like peptide type 5 | *Marsupenaeus japonicus* | 6.62E-7 |
|  | AUS\|c758128_g1 | crustin antimicrobial peptide | *Portunus trituberculatus* | 1.45E-48 |
