# Supplementary material for: Exploring the molecular basis of adaptive evolution in hydrothermal vent crab Austinograea alayseae by transcriptome analysis
Source: PLoS One. 2017 May 26;12(5):e0178417. doi: 10.1371/journal.pone.0178417 (PMC5446156; doi:10.1371/journal.pone.0178417)
Supplement: S5 Table — (DOCX) [file pone.0178417.s005.docx]

**S5 Table. Osmoregulation related genes and metal transport solute carrier (SLC) family members in the gill transcriptome of *Austinograea alayseae*.**

| **Gene category** | **Unigene ID** | **Annotation** | **Matched Organism** | **E-value** |
| --- | --- | --- | --- | --- |
| *Na^+^/K^+^-ATPase* | AUS\|c548134_g2 | sodium potassium-transporting ATPase subunit beta | *Litopenaeus vannamei* | 0 |
|  | AUS\|c513018_g1 | Na^+^/K^+^-ATPase alpha subunit | *Homarus americanus* | 2.33E-112 |
| *V-H^+^-ATPase* | AUS\|c560237_g1 | V-H*^+^*-ATPase subunit A | *Litopenaeus vannamei* | 0 |
|  | AUS\|c546756_g1 | V-type H^+^transporting ATPase subunit G | *Manduca sexta* | 6.6E-20 |
| *SLC9* | AUS\|c556616_g5 | sodium/hydrogen exchanger 9 | *Zootermopsis nevadensis* | 0 |
|  | AUS\|c561559_g1 | sodium/hydrogen exchanger | *Carcinus maenas* | 0 |
| *SLC14* | AUS\|c547063_g1 | urea transporter 2 | *Oryctolagus cuniculus* | 1.30E-13 |
| *SLC11* Proton-coupled metal ion transporter family | | | | |
| *SLC11A1* | AUS\|c686392_g1 | natural resistance-associated macrophage protein 1 | *Physeter catodon* | 1.95E-17 |
|  | AUS\|c620129_g1 | NRAMP-like transporter smf-1 | *Caenorhabditis elegans* | 1.21E-21 |
| *SLC11A2* | AUS\|c560106_g1 | solute carrier family 11 (proton-coupled divalent metal ion transporters), member 2 | *Mesocricetus auratus* | 6.77E-90 |
| *SLC30* Zinc efflux family |  |  |  |  |
| *SLC30A2* | AUS\|c562313_g1 | zinc transporter 2 | *Mus musculus* | 1.72E-88 |
| *SLC30A5* | AUS\|c562199_g1 | zinc transporter 5 | *Homo sapiens* | 3.06E-163 |
| *SLC30A6* | AUS\|c549105_g1 | zinc transporter 6-A | *Xenopus laevis* | 1.58E-83 |
| *SLC30A7* | AUS\|c560241_g2 | zinc transporter 7 | *Tribolium castaneum* | 2.62E-108 |
| *SLC30A9* | AUS\|c549880_g1 | zinc transporter 9 | *Zootermopsis nevadensis* | 1.67E-42 |
| *SLC30A10* | AUS\|c555257_g1 | zinc transporter 10 | *Mus musculus* | 1.72E-22 |
| *SLC31* Copper transporter family | | | | |
| *SLC31A1* | AUS\|c536245_g1 | high affinity copper uptake protein 1 | *Zootermopsis nevadensis* | 8.53E-56 |
|  | AUS\|c546725_g1 | high affinity copper uptake protein 1 | *Mus musculus* | 4.78E-13 |
| *SLC39* Metal ion transporter family | | | | |
| *SLC39A1* | AUS\|c554270_g1 | zinc transporter ZIP1 | *Danio rerio* | 8.16E-09 |
|  | AUS\|c559962_g1 | zinc transporter ZIP1 | *Danio rerio* | 1.87E-19 |
|  | AUS\|c562838_g2 | zinc transporter ZIP1 | *Danio rerio* | 1.13E-21 |
|  | AUS\|c550730_g1 | zinc transporter ZIP1 | *Callinectes sapidus* | 1.33E-127 |
| *SLC39A3* | AUS\|c513420_g1 | zinc transporter ZIP3 | *Homo sapiens* | 1.62E-26 |
|  | AUS\|c542164_g1 | zinc transporter ZIP3 | *Xenopus tropicalis* | 1.27E-06 |
| *SLC39A9* | AUS\|c558780_g1 | zinc transporter ZIP9 | *Homo sapiens* | 4.89E-57 |
| *SLC39A10* | AUS\|c831023_g1 | zinc transporter ZIP10-like | *Diaphorina citri* | 3.39E-09 |
|  | AUS\|c563195_g3 | zinc transporter ZIP14 | *Zootermopsis nevadensis* | 4.84E-75 |
| *SLC39A11* | AUS\|c559059_g1 | solute carrier family 39, member 11 | *Acanthisitta chloris* | 1.66E-24 |
| *SLC39A13* | AUS\|c554540_g1 | zinc transporter ZIP13 homolog | *Drosophila melanogaster* | 1.27E-06 |
