# Supplementary figures and images for: Exploring the molecular basis of adaptive evolution in hydrothermal vent crab Austinograea alayseae by transcriptome analysis
Source: PLoS One. 2017 May 26;12(5):e0178417. doi: 10.1371/journal.pone.0178417 (PMC5446156; doi:10.1371/journal.pone.0178417)

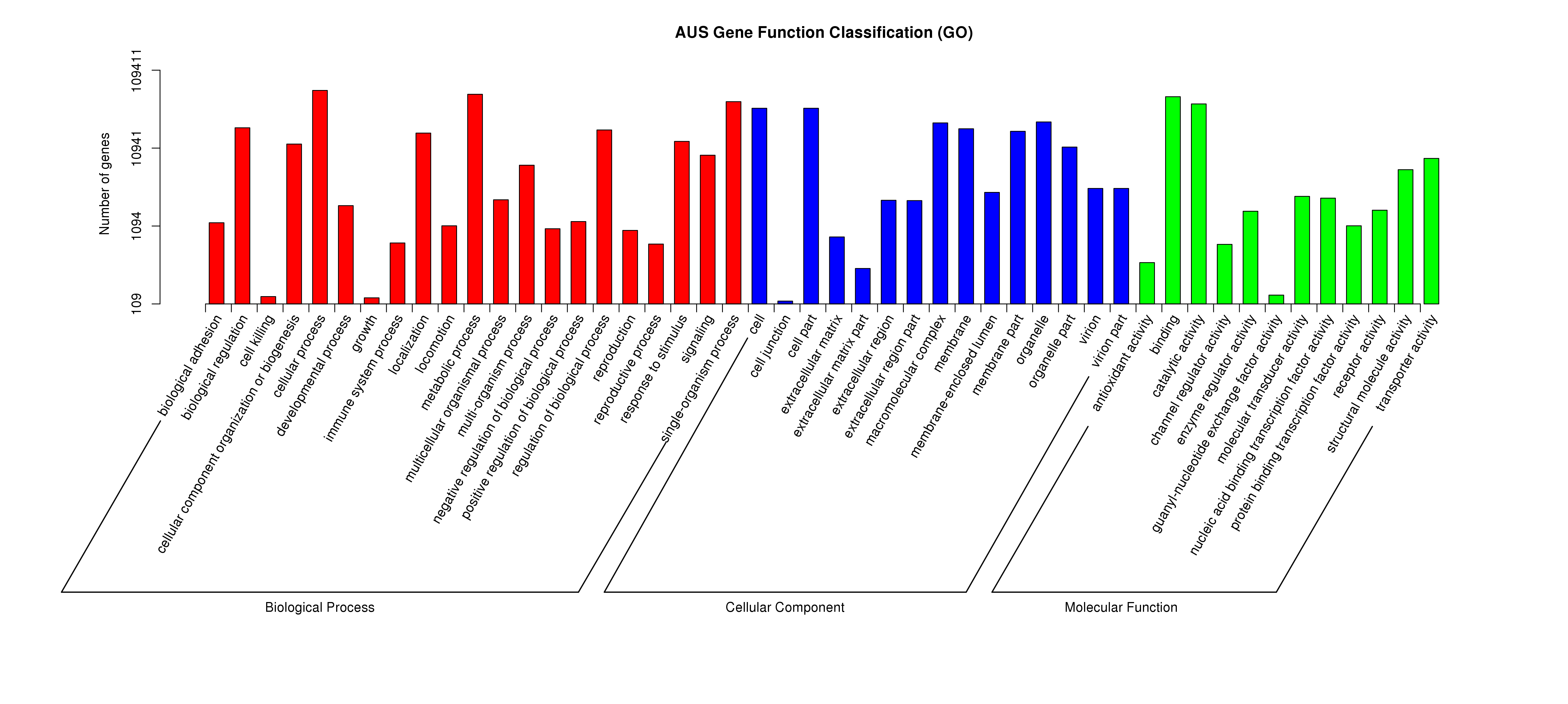

Supplement: S1 Fig — (TIF) [file pone.0178417.s006.tif]

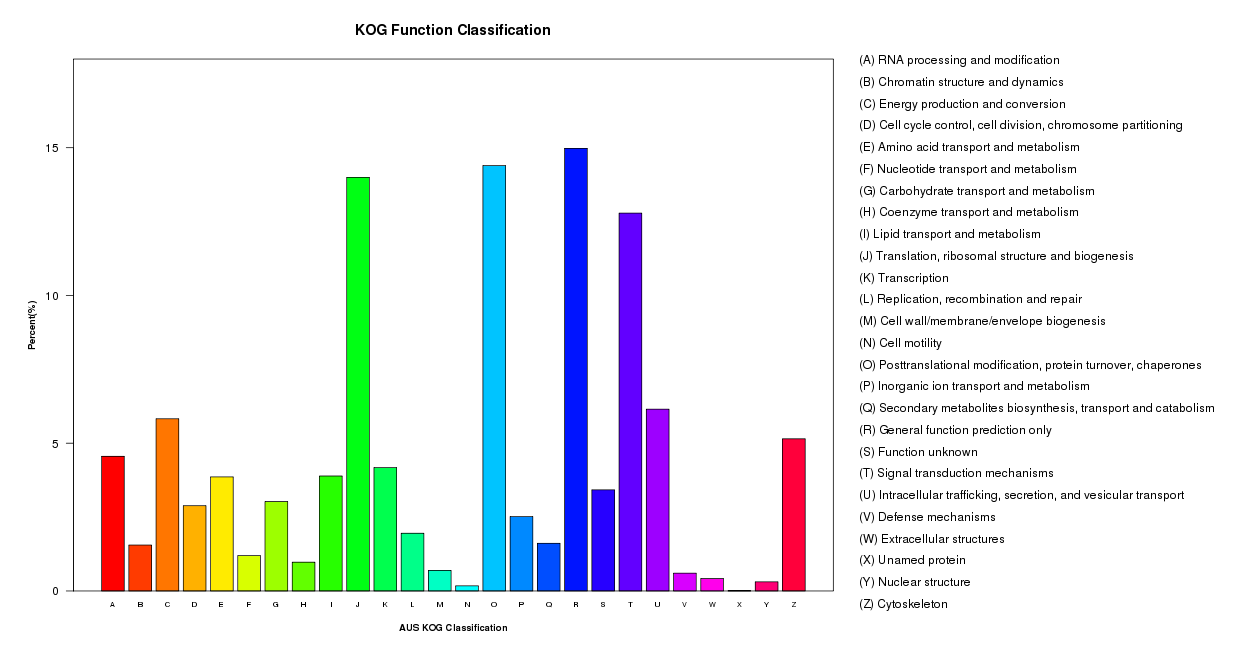

Supplement: S2 Fig — (TIF) [file pone.0178417.s007.tif]

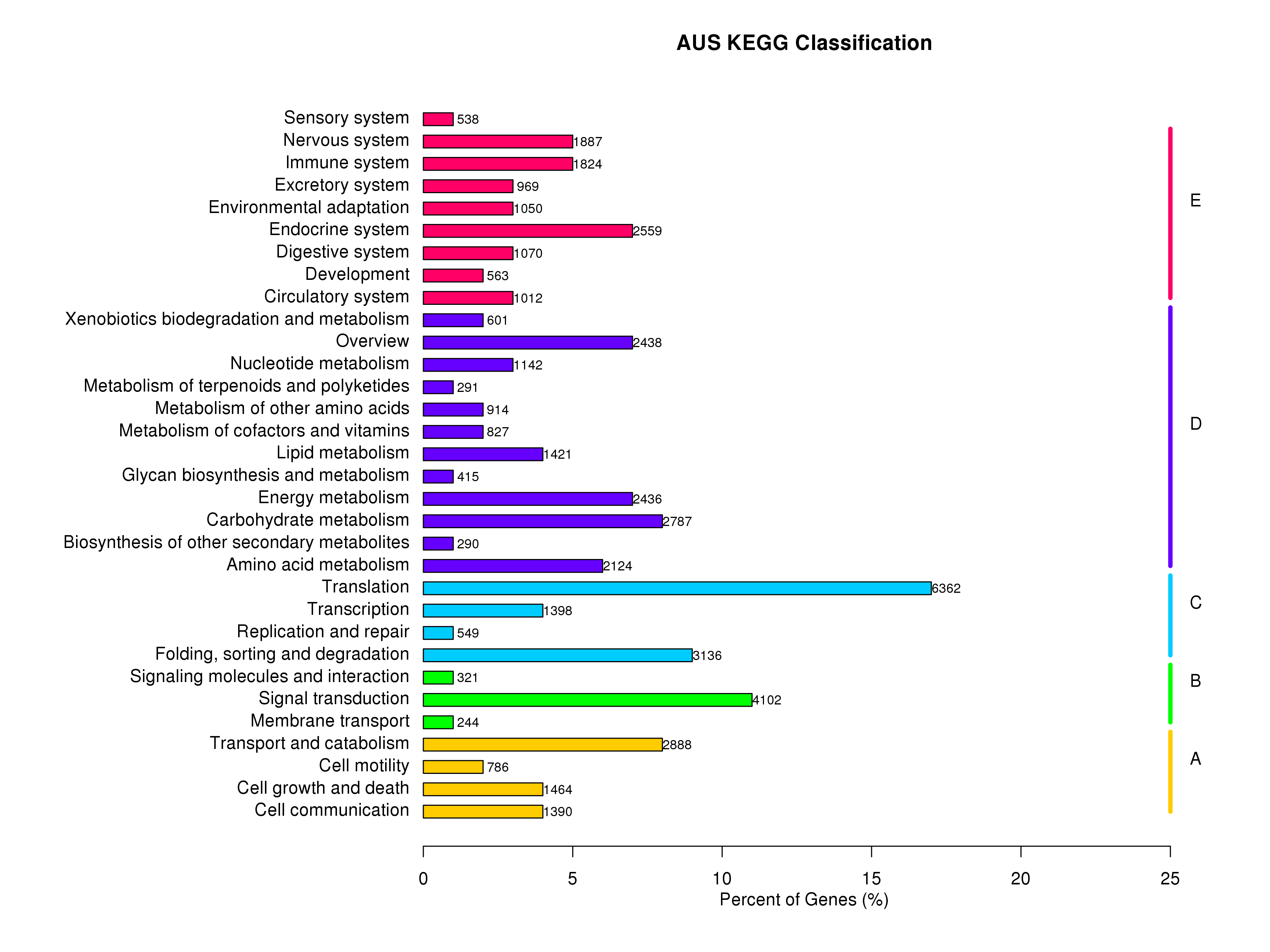

Supplement: S3 Fig — A: Cellular Processes; B: Environmental Information Processing; C: Genetic Information Processing; D: Metabolism; E: Organismal Systems. (TIF) [file pone.0178417.s008.tif]
